# When do parents report their child’s administrative ADHD diagnosis? A utilisation-based analysis from the consortium project INTEGRATE-ADHD

**DOI:** 10.25646/12676

**Published:** 2024-09-18

**Authors:** Stefan Pfeifer, Ann-Kristin Beyer, Lilian Beck, Heike Hölling, Marcel Romanos, Thomas Jans, Anne Kaman, Ulrike Ravens-Sieberer, Julian Witte, Peter Heuschmann, Cordula Riederer, the INTEGRATE-ADHD Study Group, Robert Schlack

**Affiliations:** 1 Robert Koch Institute, Department of Epidemiology and Health Monitoring, Berlin, Germany; 2 University Hospital Würzburg, Centre of Mental Health, Department of Child and Adolescent Psychiatry, Psychosomatics and Psychotherapy, Würzburg, Germany; 3 University Medical Centre Hamburg-Eppendorf, Department of Child and Adolescent Psychiatry, Psychotherapy and Psychosomatics, Research Section ‘Child Public Health’, Hamburg, Germany; 4 Vandage GmbH, Bielefeld, Germany; 5 University of Würzburg, Institute of Clinical Epidemiology and Biometry, Würzburg, Germany; 6 University Hospital Würzburg, Clinical Trial Centre, Würzburg, Germany; 7 University Hospital Würzburg, Institute for Medical Data Sciences, Würzburg, Germany; 8 DAK-Gesundheit, Hamburg, Germany

**Keywords:** ADHD, children and adolescents, epidemiological data, administrative data, utilisation

## Abstract

**Background:**

This article examines discrepancies in the frequency of diagnoses of attention-deficit/hyperactivity disorder (ADHD) in children and adolescents in Germany using information on health care utilisation from both administrative and parent-reported survey data linked at person level.

**Methods:**

5,461 parents of 0- to 17-year-olds insured with DAK-Gesundheit in 2020 and being registered with a confirmed administrative ADHD diagnosis (ICD-10 F90.0-9) in at least one quarter in 2020 (M1Q criterion) were surveyed online on their child’s ADHD diagnosis, utilisation of specialist care and therapeutic service providers. With regard to the presence of a parental report of the child’s documented ADHD diagnosis, administrative data and survey data were bi- and multivariately analysed.

**Results:**

The response rate was 21.5 %. ADHD diagnoses were given more frequently in the context of paediatric care, but in the multivariable model with the administrative data only the diagnosis made by mental health professionals (OR = 2.78), in the model with the survey data only utilisation of mental health professionals (OR = 2.99) positively predicted the parental diagnostic report. With regard to the utilisation of therapeutic service providers, only the utilisation of occupational therapy was associated with the parental report of the diagnosis in both data sources.

**Conclusions:**

Parental non-reporting of a child’s administrative ADHD diagnosis in survey studies can be in part be explained by utilisation characteristics.

## 1. Introduction

Hyperkinetic disorder (ICD-10 F90) or attention-deficit/hyperactivity disorder (ADHD; according to DSM-5) is characterised by the core symptoms of hyperactivity, impulsivity and inattention. These can vary in severity and are associated with increased individual, familial and social risks for those affected and their families, such as an increased risk of comorbidities, substance use, road traffic accidents, reduced school and educational success, lower quality of life, parenting difficulties and impairment of the parents’ relationship, as well as generally increased healthcare costs [1–6]. As one of the most frequently diagnosed behavioural disorders in children and adolescents in Germany and worldwide, ADHD, with a prevalence of around 5 % [7, 8], is also of great importance for health policy and care. For Germany, population-based prevalence data is obtained either from the claims data of the statutory health insurance funds (so-called administrative prevalence) or from primary data surveys (Infobox 1), such as the Robert Koch Institute’s epidemiological long-term study German Health Interview and Examination Survey for Children and Adolescents (KiGGS). In that study, parents were asked whether their child had ever been diagnosed with ADHD by a doctor or psychologist [8, 9]. The prevalence data and temporal trends in the frequency of diagnoses obtained in this way have varied considerably in the past [10].

**Infobox 1**

**Administrative data**

Administrative data is generated as part of administrative procedures. Important data sources for health reporting purposes are the claims data of statutory health insurance funds, from which, for example, prevalence rates (frequencies) of billed medical or psychological diagnoses can be determined. In addition, this data includes information on age and gender of the insured persons, on the utilisation of various outpatient and in patient healthcare services, drug prescription data and information on the direct costs of utilisation. The administrative diagnostic data on ADHD of children and adolescents used in the project INTEGRATE-ADHD relates to the year 2020 and stems from the statutory health insurance provider DAK-Gesundheit.

**Epidemiological data**

Epidemiological data is collected through surveys and examinations with the aim of researching the prevalence and causes of diseases in the population. Frequencies of diagnosed physical diseases and mental disorders are often assessed asking the participants whether a doctor (or a psychologist) had diagnosed the respective disease/disorder. The diagnostic data on ADHD in children and adolescents collected in the online survey of the project INTEGRATE-ADHD is based on the parents’ report of an ADHD diagnosis of their child ever made by a medical doctor or psychologist. In addition, the epidemiological data collected in the project INTEGRATE-ADHD also includes questions on sociodemographics (e.g. age and gender of the child, parental education, history of migration), psychopathology and comorbidity (e.g. ADHD symptom severity, ADHD diagnosis of the parents, anxiety, depression), risk and protective factors, quality of life, as well as satisfaction with care and barriers to utilisation.

As part of the consortium project INTEGRATE-ADHD, parents of children with an administrative ADHD diagnosis were asked about their child’s ADHD diagnosis and health care utilisation using questionnaires from the epidemiological KiGGS study and its in-depth module on child mental health, BELLA study. The parents, and from the age of 14 also the children and adolescents themselves, agreed to the subsequent linking of their survey data with their administrative data. In this way, for the first time in Germany, administrative and epidemiological ADHD diagnosis data from a parent survey can be linked at person-level [10, 11]. In that study, almost one third of parents did not report their child’s administrative ADHD diagnosis in the epidemiological survey [12]. However, parents can only report a medical or psychological ADHD diagnosis of their child in an epidemiological survey if it has been communicated to them by medical staff (doctor/patient communication) and if they are willing to disclose it. As part of the search for possible causes of the discrepancy, the present contribution deals with the question of whether and to what extent this can be explained by the specialist group of the person making the diagnosis, by specialist utilisation and by the utilisation of medical care providers. The following exploratory hypotheses were formulated:

1. If an ADHD diagnosis has been made by a mental health professional or if a mental health professional had been utilised, the probability that parents will report their child’s administrative ADHD diagnosis in the survey is increased. A guideline-based diagnosis according to the S3 guideline Attention-Deficit/Hyperactivity Disorder (ADHD) of the Association of the Scientific Medical Societies in Germany (AWMF) [13] is complex.
  In addition to diagnostic interviews with the parents and the affected child, behavioural observations, the use of ADHD-specific questionnaires and cognitive performance tests, it also involves information obtained from other people, for example the child’s teachers or educators (multi-informant principle) [13]. In addition, psychoeducation, i. e. systematic and structured education of patients and relatives about the disorder, is an integral part of guideline-compliant disease management [13]. According to the guideline, the diagnosis should be made either by a specialist in child and adolescent psychiatry and psychotherapy or a child and adolescent psychotherapist or a psychological psychotherapist with additional qualifications for children and adolescents or a specialist in paediatric and adolescent medicine with experience and expertise in the diagnosis of ADHD [13]. These specialist healthcare providers are referred to below as mental health professionals and their utilisation as utilisation of mental health professionals. It can be expected that those parents whose child received an ADHD diagnosis by a mental health professional are more likely to have been informed of their child’s diagnosis (reduction of a reporting bias due to a lack of doctor-patient communication). It can also be assumed that parents and affected children are more likely to receive psychoeducation, which could increase the acceptance of the diagnosis (reduction of social-desirability bias) and in turn increase the likelihood of a parental report. Further, it can be expected that the diagnostic effort is higher in a setting specialised on mental health, which means that parents remember the diagnosis better (reduction of recall bias) and are therefore more likely to report it.
2. When utilising remedies such as occupational or speech therapy, the probability of a parent report of an administratively documented ADHD diagnosis of the child is increased. The prescription of remedies is often part of a guideline-based multimodal therapy approach [13]. It can therefore be assumed that the utilisation of remedies in the presence of an ADHD diagnosis contributes to improved awareness of the diagnosis among parents and thus indirectly to the fact that parents are more likely to report their child’s diagnosis in the epidemiological survey.

In the project INTEGRATE-ADHD, information on the utilisation of medical specialists and the utilisation of medical care providers is available at person-level from both the administrative data and the information provided by parents in the online survey. This makes it possible to analyse this information from both data sets comparatively and with a view to its predictive ability for the existence of a parental report of the child’s administrative ADHD diagnosis. Information on who made the child’s ADHD diagnosis is available in the administrative data and only for the children and adolescents who were newly diagnosed in 2020. These are referred to below as the incidence cohort. The analyses of this data are compared with the analyses of specialist utilisation from the online survey data for the entire sample. Utilisation of therapeutic providers are analysed both from the administrative data for the incidence cohort and for all children and adolescents from the online data.

**ADHD in Germany – Comparison and integration of administrative and epidemiological ADHD diagnostic data through clinical assessment (INTEGRATE-ADHD)**

**Consortium partners:** Robert Koch Institute Berlin, Department of Epidemiology and Health Monitoring, Germany; University Hospital Würzburg, Department of Child and Adolescent Psychiatry, Psychosomatics and Psychotherapy, Germany; University Medical Centre Hamburg-Eppendorf, Department of Child and Adolescent Psychiatry, Psychotherapy and Psychosomatics, Research Section ‘Child Public Health’, Germany; Vandage GmbH, Germany; University of Würzburg, Germany, Institute for Clinical Epidemiology and Biometry, Germany; DAK-Gesundheit, Germany

**Data holder:** Robert Koch Institute

**Objectives:** Identification of potential causes for the discrepancies between administrative ADHD diagnostic data (based on health insurance claims data) and epidemiological ADHD diagnostic data (based on surveys) for Germany, integration and validation of these data through a guideline-based clinical examination

**Study design:** Cross-sectional online survey, additional clinical examination of a sub-sample, data linkage with administrative health insurance data

**Population:** Children and adolescents who were insured with DAK-Gesundheit in 2020 and who were 0 to 17 years old at that time and for whom an administrative ADHD diagnosis labelled as confirmed was available in at least one quarter

**Gross sample**: 24,880 children and adolescents insured with DAK-Gesundheit with an administrative ADHD diagnosis

**Net sample:** 5,461 surveyed parents, 202 clinically examined children and adolescents

**Data collection period:** October 2021 to August 2022 (online survey), January 2022 to January 2023 (online clinical examination)

More information in German at www.rki.de/integrate-adhd

## 2. Methods

### 2.1 Study design and conduct

INTEGRATE-ADHD is designed as a cross-sectional interview and examination study of parents (respondents) of children and adolescents (target persons) insured with DAK-Gesundheit, the third largest statutory health insurance company in Germany. Children and adolescents were included if they had a confirmed outpatient or inpatient ADHD diagnosis (ICD-10 F90.0-9, primary or secondary diagnosis) in at least one quarter in 2020 (M1Q criterion) and if they were 0 to 17 years old at that time.

Of a total of 848,110 children and adolescents insured with DAK-Gesundheit in 2020, 24,880 (gross sample) were selected according to the inclusion criteria and their parents were invited to participate in the study. In the invitation letter, parents were informed that children with and without an ADHD diagnosis would be compared and that their participation was important even if they were not aware of a child’s ADHD diagnosis. A total of 5,919 parents took part in the online survey. The online survey was conducted from October 2021 to August 2022 using adapted questionnaires from the nationwide epidemiological KiGGS study [14–16] and from its in-depth module on child mental health, the BELLA study [17, 18]. After excluding 458 people for formal or substantial reasons (e.g., more than 50 % missing values or inconsistencies concerning age or sex between the administrative and epidemiological data set), the net sample was 5,461 participants. The response rate according to the American Association for Public Opinion Research (AAPOR’s) Standard Definitions, Version 9 (RR3) was 21.5 % [19]. In addition, a subsample of children and adolescents whose parents had participated in the online survey took part in an online clinical assessment in accordance with the German AWMF S3 guideline ADHD [13]. Subsequently, the data from the online survey, the administrative data and the clinical data were linked at the person level to form an integrated data set in accordance with the principles of the manual ‘Good Practice Data-Linkage’ [20]. The data from the clinical sample are not the subject of this study. For details on study design and conduct as well as sampling, see [10, 11].

#### Representativeness of the sample

The children and adolescents insured with DAK-Gesundheit can be considered approximately representative of the population of children and adolescents in Germany in terms of gender and age [11]. With regard to the population of children and adolescents with an administrative ADHD diagnosis, comparisons of the INTEGRATE-ADHD gross sample with nationwide outpatient ADHD diagnostic data from the Central Research Institute of Ambulatory Health Care in Germany (Zi) from 2015 and 2016 [21] showed only very slight deviations in terms of distribution by gender, while younger children were over- and older children and adolescents were underrepresented in the INTEGRATE-ADHD gross sample [11].

#### Definition of the incidence cohort and identification of the person diagnosing the disorder (diagnostician)

In the case of prevalent (pre-existing) ADHD diagnoses, it is not always possible to clearly identify the person who made the diagnosis, as since 2005 it has been technically possible to transfer permanent diagnoses from a previous quarter to the following quarter in German medical surgery management systems [22]. In order to identify the diagnostician as clearly as possible, the administrative data was restricted to children and adolescents with an administrative ADHD diagnosis firstly documented in 2020 (hereafter also referred to as the incidence cohort). In the project INTEGRATE-ADHD, administrative data of the target persons is available for the insurance years 2019 and 2020. Children and adolescents were defined as incident if they had a diagnosis-free pre-observation period of at least four quarters.

The specialist group of the diagnostician was determined as follows: in the administrative data, diagnoses are documented on a quarterly basis together with a case identification number (case ID) that can be clearly assigned to an insured person. A Doctor’s ID can in turn be assigned to the case ID via the pseudonymised German lifelong physician’s number (LANR; in German: Lebenslange Arztnummer), which contains a specialist group code. Several medical specialists or therapists (treating or diagnosing) can be involved in a treatment case. A unique assignment of the diagnostician is possible if only one doctor ID is documented for a case ID. In addition, a doctor ID and case ID were assigned if the case had a short duration (difference between case start and case end < five days). In this way, the diagnostician could be identified in the administrative data for a total of 93 % of children and adolescents with an incident ADHD diagnosis.

**Figure 1:**
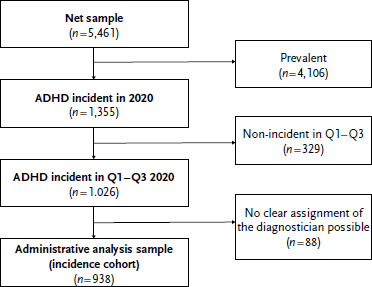
Flowchart of the administrative analysis sample (children and adolescents with an incident ADHD diagnosis (ICD-10 F90.0-9) in 2020 insured with the statutory health insurance provider DAK-Gesundheit in quarters one to three in 2020). Source: Own depiction ADHD = attention-deficit/hyperactivity disorder

As a follow-up period for the analysis of the utilisation of treatment providers, one quarter after the documentation of the incident ADHD diagnosis was defined. Thus, only cases from the quarters one to three 2020 (*n* = 938) were included in the analysis of the incidence cohort (Figure 1).

#### Formation of the analysis groups of diagnosticians and therapeutic service providers

Four analysis groups were formed on the basis of the specialist group codes of the German lifelong physician’s number (LANR) [23] (Infobox 2):

1. Mental health professionals (psychiatric, psychological, or psychotherapeutic service providers; neuropaediatricians were also assigned to this group).
2. General practitioners
3. Paediatricians
4. Other physicians.

**Infobox 2**

**Group categorisation of diagnosticians using the German lifelong physician’s number (LANR; administrative data)**

1. Mental health professionals
  ► General practitioner neuropaediatrics/child neuropsychiatry (LANR 38)
  ► Specialist neuropaediatrics/child neuropsychiatry (LANR 44)
  ► Child and adolescent psychiatry and psychotherapy (LANR 47)
  ► Neurology and psychiatry (LANR 51)
  ► Psychiatry und psychotherapy (LANR 58)
  ► Forensic psychiatry (LANR 59)
  ► Psychosomatic medicine und psychotherapy (LANR 60)
  ► Medical psychotherapist (LANR 61)
  ► Psychological psychotherapist (LANR 68)
  ► Child and adolescent psychotherapist (LANR 69)
2. General practitioners
  ► General practitioner (LANR 1)
  ► General practitioner without specialist training (LANR 2)
  ► General practitioner (internist) (LANR 3)
3. Paediatrics and adolescent medicine
  ► General paediatrics and adolescent medicine (LANR 34)
  ► Specialist paediatrics and adolescent medicine (LANR 40)
  ► Paediatrics and adolescent medicine with a focus on primary care/specialist care (LANR 46)
4. Other physicians
  ► All service providers that do not fall into one of the other categories

Two groups were formed based on the German therapeutic service code [24] to analyse the utilisation of therapeutic service providers:

1. Occupational therapists (X5)
2. Speech therapists and breathing, speech and voice coaches (X3).

#### Definition of the analysis sample from the online survey data

The online survey data was used for the epidemiological analyses (*n* = 5,461). Parental information on their child’s ADHD diagnosis was available for a total of 5,211 target persons.

### 2.2 Instruments

#### Parent-reported ADHD diagnosis

Based on the questionnaires of the KiGGS study [8, 9], parents were asked about their child’s ADHD diagnosis: ‘Has your child ever been diagnosed with attention-deficit/hyperactivity disorder, also known as ADHD or ADD?’, with the answer options ‘Yes’, ‘No’, ‘Don’t know’. If the parents answered this question in the affirmative, they were asked by whom the diagnosis was made. Response options were ‘doctor’, ‘psychologist’ and ‘other’. According to the KiGGS ADHD case definition [8, 9], cases were considered valid if the diagnosis was made by a doctor or a psychologist or if information was provided on institutions in which the diagnosis could reasonably be assumed to have been made by medical or psychological staff (e.g. ‘university clinic’, ‘child and adolescent psychiatry’, ‘social paediatric centre’, etc.).

#### Utilisation of specialist care and therapeutic service providers

In the online survey, the utilisation of specialist medical care was recorded retrospectively with the following item from the KiGGS study: ‘Please tell us which of the following registered doctors you have seen for your child in the past 12 months (and how often)’. Response options were: ‘Paediatrician’, ‘General practitioner’, ‘Psychiatrist, Child and adolescent psychiatrist, Medical psychotherapist’ and ‘Psychologist, psychological psychotherapist’ and ‘My child has NOT utilised a registered doctor in the past 12 months’. Utilisation (per specialist group) was counted valid if parents reported at least one utilisation in the past twelve months. To achieve comparability with the group classification for diagnosticians in the administrative data, the categories ‘psychiatrist, child and adolescent psychiatrist, medical psychotherapist’ and ‘psychologist, psychological psychotherapist’ were combined into a single group ‘mental health professionals’. The utilisation of occupational and speech therapies was assessed using the item from the KiGGS study: ‘Which of the following therapists have you utilised for your child in the past 12 months and how often?’ Response options were ‘Occupational therapist’, ‘Speech and language therapist’ and the option ‘My child has NOT utilised a therapist in the past 12 months’. Again, utilisation was counted valid if it was reported at least once in the past twelve months.

#### Confounders

The following confounders were included in the multivariate analyses:

► Age of the child at the time of the survey (in years).
► Gender (female, male). The administrative data contained only binary gender information; in the online survey, the gender of 27 respondents was reported as ‘other’. As this group was too small to be statistically analysed, the individuals were assigned the gender from the administrative data.
► Parental education according to the CASMIN (Comparative Analysis of Social Mobility in Industrial Nations) classification (low, medium, high) [25]; categorisation was based on the person with the highest level of education in the household.
► Migration background of the child (operationalised according to [26]). The concept of ‘migration background’ has recently been criticised for not being sufficiently diverse [27]. Instead, it is recommended to stratify analyses according to individual variables such as country of birth, nationality, residence status or language skills. However, this is not possible in the present study due to insufficient case numbers.
► Urbanicity (urban versus rural region) according to the INKAR (‘Indikatoren und Karten zur Raum- und Stadtentwicklung’) classification of the Federal Institute for Research on Building, Urban Affairs and Spatial Development [28].
► Density of care: regional ratio of (specialist) physician per 100,000 inhabitants in the spatial planning region of the child’s place of residence in terms of child and adolescent psychiatrists, medical psychotherapists, paediatricians and general practitioners [29], applied as quintile values. The quintiles were calculated separately for the administrative sample and the epidemiological sample.
► Prescription rate of ADHD medication (person received at least one ADHD-specific medication prescription in 2020, defined as ATC codes N06BA04, N06BA09, N06BA02, N06BA12, N06BA21)
► Parent-rated health status of the child (very good/good, fair, poor/very poor) [30].
► ADHD symptom severity, recorded using the ADHD external assessment form (FBB-ADHS-V or FBB-ADHS [31]).

### 2.3 Statistical analysis

Bivariate group comparisons of categorical variables were conducted using weighted non-adjusted logistic regressions. For multivariate analysis, three binary logistic regression models, adjusted for the confounders, were each calculated for both the incidence cohort (administrative data) and the online survey sample. The dependent variable in each of the models was the presence of a parent-report of the child’s administrative diagnosis of ADHD (yes/no). For the incidence cohort, in the first model only the specialist group of the diagnostician was included as an independent variable (in addition to the confounders). In the second model, in addition to the confounders only the utilisation of therapeutic service providers from the administrative data in 2020 was included (coding: yes/no). Finally, the third model included both the diagnostician, the utilisation of therapeutic service providers and the confounders simultaneously. The same procedure was applied for the online sample. In the first model, only parent-reported utilisation of medical specialists in the past twelve months (coded yes/no) was included as an independent variable in addition to the confounders; in the second model, only parent-reported utilisation of therapeutic service providers in the past twelve months (coded yes/no) was included; in the third model, both utilisation of medical specialists and utilisation of therapeutic service providers plus the confounders were included. Group differences with *p* < 0.05 were considered statistically significant. The effect sizes of the predictors in the logistic models are expressed as odds ratios (OR).

#### Weighting

Deviations of the net sample from the gross sample were adjusted using population weights, which normalise the net sample to the gross sample [11]. The population weights are determined by the inverse probability of an individual participating in the study; individuals with a low probability of participation represent a larger portion of the population than those with a high probability of participation. To determine the participation probabilities, a logistic regression model was calculated based on administrative data. The model included the child’s age in 2020, the German Index of Socioeconomic Deprivation (GISD), the ADHD incidence rate in 2020, the prescription rate of ADHD medication, whether the child received at least one outpatient behavioural therapy, whether the child had outpatient contact with mental health professionals, and whether the child’s initial ADHD diagnosis was made in general practice or in paediatric care if the information could be assigned. Analyses were weighted and conducted with the Stata 17.0 software package using the svy-procedure.

## 3. Results

###  

#### Descriptive statistics – Incidence cohort (administrative data)

The mean age of the children and adolescents in the incidence cohort (*n* = 938) was 11.2 years, the age range was 2 to 19 years and the proportion of boys was 71.7 % (Table 1). The age group 7 to 10 years was the most populous with 40.8 %. Among the parents of children and adolescents in the incidence cohort, 55.1 % reported an administrative diagnosis of ADHD for their child. The proportion of parents in the low education group was 9.5%. The proportion of children and adolescents with a migration background was 6.7 %. The mean ADHD symptom severity was *M* = 1.2, and the prescription rate for ADHD medication was 22.2 %. The parent-rated health status of the child was good or very good in 90.8 % of the children. Overall, 61.3 % of the children and adolescents were living in urban areas. The level of care for diagnosis-incident children and adolescents in the administrative data was at the mean value of the expected density of care for the incidence cohort (*M* = 3.0) with regard to medical-psychotherapeutic care (excluding child and adolescent psychiatric care), paediatric care and general practitioner/general practitioner care. In contrast, the density of child and adolescent psychiatric care was below average (*M* = 2.8).

#### Descriptive statistics – Online sample

In the online sample (*n* = 5,461), the mean age was 12.6 years (age range: 2 to 19 years) and the proportion of boys was 74.1 %. The age group with the highest proportion of boys was 14 to 17 years with 33.4 %. Among the parents of children and adolescents in the online sample, 71.6 % reported an administrative ADHD diagnosis for their child, see also [12]. The proportion of parents with a low level of education was 10.4 % (Table 1), and the proportion of children with a migration background was 6.5 %. The prescription rate for ADHD medication was 42.7 %, and the proportion of children and adolescents living in urban areas was 63.6 %. The parent-rated health status of the child was good or very good in 86.3 % of the children (Table 1). The mean quintile values for density of care were 3.0 for medical/psychotherapeutic care (excluding child and adolescent psychiatric care), 3.0 for paediatric care and also 3.0 for general practitioner/general medical care. These values correspond to the expected mean values for the online sample. The lower mean value for paediatric and adolescent psychiatric care (*M* = 2.8), on the other hand, indicates a below-average care density in comparison.

#### Frequency of diagnosticians and utilisation of therapeutic service providers – Incidence cohort (administrative data)

Every second diagnosis of ADHD in a child or adolescent was made in paediatric care, and about one in three by a mental health professional (Table 2). Just under one in ten ADHD diagnoses were made by general practitioners. ADHD diagnoses made in the context of inpatient care or by other medical specialists played only a minor role in quantitative terms.

Significant differences by the availability of a parent report of the child’s administrative ADHD diagnosis are particularly evident for those diagnosed by paediatricians and mental health professionals (Table 2). Among non-reporting parents, the proportion of ADHD diagnoses made by paediatricians is 16.5 percentage points or 39.8 % higher than among reporting parents, while the proportion of ADHD diagnoses made by mental health professionals is 13.3 percentage points or 32.5 % lower among non-reporting parents than among reporting parents.

With regard to the utilisation of therapeutic service providers in the administrative data, occupational therapy was the most commonly used service, accounting for about one fifth (Table 2). There were no statistically significant differences between parents who reported and those who did not report their child’s administrative diagnosis of ADHD. Speech therapy was only used by just under one in fourteen children or young people. Again, there were no significant differences between parents who did and did not report a diagnosis for their child.

#### Frequencies of utilisation of specialist medical care and utilisation of therapeutic service providers – Online sample

Just under two-thirds of parents surveyed reported utilisation of a paediatrician in the past twelve months (Table 3). Just over half of respondents reported utilisation of mental health professionals, and just over a third reported utilisation of general practitioners.

There were significant differences in the frequencies of utilisation of specialist medical care: while the utilisation of paediatricians care was significantly more common among parents who did not report an administrative diagnosis of ADHD for their child, mental health professionals were more commonly utilised by parents who did report a diagnosis for their child. The frequency of utilisation of general practitioners did not differ between the two groups.

Approximately one-sixth of children and adolescents were reported to utilise occupational therapists in the past twelve months, and approximately one in thirteen children received speech therapy (Table 3). Occupational therapy was significantly more frequently reported by parents who reported a child’s diagnosis. Speech therapy was reported significantly less frequently by parents who reported epidemiologically an ADHD diagnosis for their child than by parents who did not report a child’s diagnosis.

#### Multivariate analyses – Incidence cohort (administrative data)

The results of the multivariate analyses adjusted for the confounders confirm the findings of the descriptive analyses. A parent-report of the child’s ADHD diagnosis was almost three times more likely when the diagnosis was made by a mental health professional when – besides the confounders - only medical specialists were considered in the model (Table 4). When considering only the utilisation of therapeutic service providers, neither type of utilisation is significantly associated with the presence of a parent report of an ADHD diagnosis (Table 4). The findings from the models 1 and 2 were confirmed when specialist diagnosis and the utilisation of remedial services were considered simultanously (Table 4).

#### Multivariate analyses – Online sample

In the multivariate analysis of the online survey data, a parent report of the child’s ADHD diagnosis is 31 % less likely to predict paediatric utilisation when - in addition to the confounders - only the utilisation of medical specialists in the past twelve months were considered in the model. In contrast, in this model a parental report is three times more likely if a mental health professional had been utilised in the past twelve months. The utilisation of a general practitioner is significantly less likely to predict a parent report (Table 4). When only the utilisation of therapeutic service providers in the past twelve months was considered, the utilisation of occupational therapy was significantly associated with a 1.5-fold increase in the odds of a parental report of ADHD, while there was no significant association with the utilisation of speech therapy (Table 4). When the utilisation of medical specialists and the utilisation of therapeutic service providers were considered simultaneously, the estimates for utilisation of medical specialists and occupational therapists remained significant at the same or similar levels (Table 4).

## 4. Discussion

As part of the INTEGRATE-ADHD data linkage project, the probability of a parental report of a present administrative diagnosis of ADHD (ICD-10 F90.0-9) in children and adolescents was examined as a function of specialist diagnosis, utilisation of medical specialists or therapeutic service providers, respectively, both on the basis of health insurance claims data and on the basis of a survey of parents. As the diagnosing specialist can only be reliably identified for new diagnoses of ADHD, the analysis of the administrative data was limited to incident cases in 2020. It had been expected that the parent report would be more predictive if the diagnosis was made by a mental health professional or if a mental health professional was utilised in the past twelve months, and if occupational and/or speech therapy had been received according to the claims data or according to the parental report in the epidemiological survey.

According to the administrative data, ADHD diagnoses in children and adolescents were most frequently made by paediatricians. Among children without a parental report of their ADHD diagnosis, the proportion of ADHD diagnoses made by paediatricians was significantly higher, while the proportion of diagnoses made by mental health professionals was lower in this group. Similarly, in the multivariate analysis of the administrative data, a parental report was predicted only by diagnoses made by mental health professionals. The result was similar when analysing the online sample. There, the utilisation of paediatricians was evenly associated with a lower likelihood of a parent report. The results might suggest that when a diagnosis is made by a mental health professional or when mental health professionals are utilised, the diagnosis is more likely to be discussed with the parents and those affected (effect of doctor-patient communication) and affected individuals and their families are more likely to receive psychoeducation. This in turn may contribute to an improved recall (i.e., reduction of recall bias) and a better acceptance of the diagnosis by the parents and, grace to reduced social desirability effects, to a higher likelihood of a parental reporting of the diagnosis. Although these hypotheses cannot be directly confirmed with the data of INTEGRATE-ADHD, the results do not contradict them either. It cannot be addressed by this study whether or not parental non-reporting was present despite extensive diagnostics and a communication of the diagnosis by the health care providers, for example because the parents did not accept the ADHD diagnosis of their child or feared social stigma. Thus, more research is needed in this area.

If doctor-patient communication, the success of which is an important factor in coming to terms with the diagnosis [32], is important for the parents’ reporting of their child’s ADHD diagnosis, time and economic constraints may also play a role. The majority of ADHD diagnoses are made in an outpatient setting, as evidenced by the administrative data. In everyday practice, there is often little time for more indepth patient consultations, with an average of less than eight minutes available for each patient contact [33]. In recent years, structural deficits in the health care of children and adolescents have been repeatedly pointed out, such as regional undersupply and staff shortages, especially in the outpatient sector [34, 35]. It can therefore be assumed that care deficits and a lack of human resources are relevant barriers to both time-intensive, guideline-based ADHD diagnosis and adequate doctor-patient communication in this area.

Parents were also more likely to report their child’s administrative diagnosis of ADHD if, according to the survey data (online sample), their child had utilised occupational therapy in the past twelve months prior to the survey date. The same finding was present in the administrative data, although not statistically significant. Along with conditions of the central nervous system and developmental delay, ADHD is one of the three most common indications for occupational therapy prescriptions [36]. It is therefore reasonable to assume that parents whose child has an administrative diagnosis of ADHD and is receiving occupational therapy are more likely to be aware of the ADHD diagnosis and that their recall of the diagnosis is increased as a result. Both are prerequisites for parents to be able to report the diagnosis. However, recall bias can never be completely ruled out [37]. The fact that recall bias plays a fundamental role in parental reporting of an ADHD diagnosis is demonstrated by the results of several studies [38, 39]. For example, in the Dunedin Multidisciplinary Health and Development Study, only 22 % of parents of adults with a confirmed childhood diagnosis of ADHD were able to correctly recall an ADHD diagnosis of their child before the age of twelve years, while 78 % had no recollection [38]. However, the recall period there was considerably longer at a minimum of 26 years compared to a maximum of 32 months in the project INTEGRATE-ADHD. Recall bias is therefore likely to be less important in the present study.

INTEGRATE-ADHD is one of a series of recent studies on the consistency of epidemiological diagnosis data with routine statutory health insurance (SHI) data, which documents the increasing interest in this topic. In a study of 6,588 adults insured by Barmer-GEK, Vogelgesang et al. found that respondents’ diagnoses of depression and any mental disorder were less consistent with administrative data than, for example, diabetes or hypertension [40]. A Belgian meta-analysis shows that the predictive value of administrative data for actual diagnoses also varies according to the type of mental disorder [41]. However, both studies are based on adult data and self-reports. Comparable studies for childhood and adolescence and for proxy reports are still rare.

The present study has a number of limitations. The first is the time lag between the administrative data (data base was 2020) and the online survey data (survey period October 2021 to August 2022). This was a minimum of nine months (31 December 2020 to the start of the online survey in early October 2021) and a maximum of 32 months (1 January 2020 to the end of the survey in August 2022) and could have affected respondent recall. However, the administrative data were made available by the consortium partner DAK-Gesundheit only nine months after the end of the insurance year 2020, which can be considered extraordinarily fast. It would be difficult to achieve greater temporal proximity between administrative and epidemiological survey data, which is, in turn, a major strength of this study. The comparability of the results of the analyses of the two data sets also suggests that the time lag does not affect their validity. Finally, the project INTEGRATE-ADHD provides for the first time conjunctive, i.e., linked administrative and epidemiological ADHD diagnostic data at the person-level. Another strength of this study is that, after weighting, the study data can be considered to be approximately representative of the population. However, the weighting does not take into account the specific ‘not missing at random’ non-response that occurs when parents of children with ADHD do not participate because they are sure their child has ADHD, or when parents who are sure their child does not have ADHD do not participate.

In conclusion, differences in the likelihood of parents reporting a child’s administrative diagnosis of ADHD in an epidemiological survey can be attributed, at least in part, to health care-related characteristics that could be quantified in the present study. However, the reasons why parents did not report their child’s administrative diagnosis of ADHD – inadequate or lack of doctor-patient communication, lack of recall or fear of stigma (social desirability) – must ultimately remain open, as this cannot be clearly answered on the basis of the INTEGRATE-ADHD data. However, it can be assumed that the number of parents who are unaware of their child’s administratively documented ADHD diagnosis is high. This points to the need for better doctor-patient communication, possibly a lack of psychoeducation, but may also raise questions about the validity of the diagnosis or documentation in such cases. As part of INTEGRATE-ADHD, these questions will be addressed in further analyses using the guideline-based ADHD diagnostic procedures carried out on the clinical sub-sample.

## Key statement

- About half of the incident ADHD diagnoses were documented in the context of paediatric care, one third in the context of psychiatricpsychological-psychotherapeutic care.
- Among parents who did not report their child’s ADHD diagnosis, the proportion of ADHD diagnoses made by paediatricians was 40 % higher than among parents who reported the diagnosis.
- Parents were more likely to report their child’s ADHD diagnosis if their child had utilised occupational therapy.
- There are differences in the likelihood of parent reports of their child’s administrative ADHD diagnosis by who made the diagnosis, the utilisation of medical specialists and the utilisation of therapeutic service providers.

## Figures and Tables

**Table 1:** Descriptive statistics. Source: INTEGRATE-ADHD

	Incidence cohort (administrative data)^a^				Online sample^b^			
	*n*	%	(95 % CI)	Mean (SE)	*n*	%	(95 % CI)	Mean (SE)
**Gender**								
Girls	260	28.3	(25.5 – 31.4)	–	1,386	25.9	(24.7 – 27.1)	–
Boys	678	71.7	(68.6 – 74.5)	–	4,075	74.1	(72.9 – 75.3)	–
**Mean age^c^**								
	938	–	–	11.2 (0.1)	5,461			12.6 (< 0.1)
**Age groups^c^**								
0 – 2 years	1	0.1	(0.0 – 0.8)	–	3	< 0.1	(0.0 – 0.2)	–
3 – 6 years	63	7.1	(5.6 – 9.0)	–	167	3.5	(3.0 – 4.0)	–
7 –10 years	399	40.8	(37.7 – 44.0)	–	1,351	24.3	(23.1 – 25.4)	–
11 –13 years	242	24.5	(21.8 – 27.3)	–	1,827	31.2	(30.0 – 32.5)	–
14 –17 years	193	22.2	(19.5 – 25.1)	–	1,770	33.4	(32.2 – 34.8)	–
18 –19 years	40	5.3	(3.9 – 7.1)	–	343	7.5	(6.8 – 8.3)	–
**Parent-reported ADHD diagnosis of the child**								
Present	518	55.1	(51.7 – 58.4)	–	3,947	71.6	(70.3 – 72.9)	–
Not present	364	44.9	(41.6 – 48.3)	–	1,264	28.4	(27.1 – 29.7)	–
**Parental education (CASMIN)^d^**								
Low	86	9.5	(7.7 –11.6)	–	560	10.4	(9.6 –11.3)	–
Medium	561	62.8	(59.5 – 66.0)	–	3,271	63.2	(61.9 – 64.5)	–
High	253	27.7	(24.8 – 30.7)	–	1,355	26.4	(25.1 – 27.6)	–
**Migration background (two-sided** ^e^ **)**								
No	854	93.3	(91.4 – 94.8)	–	4,948	93.5	(92.7 – 94.1)	–
Yes	59	6.7	(5.2 – 8.6)	–	332	6.5	(5.9 – 7.3)	–
**Symptom severity**								
FBB-ADHD scale	922	–	–	1.2 (< 0.1)	5,364	–	–	1.2 (< 0.1)
**Prescription rate of ADHD medication**								
Yes	273	22.2	(19.8 – 24.7)	–	2,788	42.7	(41.4 – 44.1)	–
No	665	77.8	(75.3 – 80.2)	–	2,673	57.3	(55.9 – 58.6)	–
**Parent-rated health status of the child**								
Very good/good	848	90.8	(88.7 – 92.5)	–	4,689	86.3	(85.3 – 87.2)	–
Average	85	8.7	(7.0 –10.7)	–	703	12.6	(11.7 –13.5)	–
Poor/very poor	5	0.5	(0.2 –1.3)	–	62	1.1	(0.9 –1.5)	–
**Urbanicity**								
Urban	572	61.3	(58.1 – 64.5)	–	3,431	63.6	(62.3 – 64.9)	–
Rural	355	38.7	(35.5 – 41.9)	–	1,949	36.4	(35.1 – 37.7)	–
**Density of care^f^**								
Medical psychotherapist	927	–	–	3.0 (< 0.1)	5,380	–	–	3.0 (< 0.1)
Child and adolescent psychiatrist	927	–	–	2.8 (< 0.1)	5,380	–	–	2.8 (< 0.1)
Paediatrician	927	–	–	3.0 (< 0.1)	5,380	–	–	3.0 (< 0.1)
General practitioner	927	–	–	3.0 (< 0.1)	5,380	–	–	3.0 (< 0.1)

*n* = unweighted, % = weighted, CI = confidence interval, SE = standard error, ADHD = attention-deficit/hyperactivity disorder, CASMIN = Comparative Analysis of Social Mobility in Industrial Nations, FBB = proxy rating (‘Fremdbeurteilungsbogen’)

^a^Incident cases in quarters one to three in 2020 in which the diagnostician was clearly assignable (*n* = 938)

^b^Epidemiological online survey

^c^At the time of the survey

^d^Person with highest level of education in the household

^e^Two-sided: migration background of the mother and father

^f^Mean values of the quintiles of the regional ratios of the respective specialist group per 100,000 inhabitants in the spatial planning region of the child’s place of residence

**Table 2:** Frequencies of specialist diagnoses and utilisation of therapeutic service providers in administrative data (incidence cohort), overall and by presence of parent-reported ADHD diagnosis. Source: INTEGRATE-ADHD

	Incidence cohort (administrative data)									
				Parent-reported ADHD diagnosis						
	Total			Present			Not present			
	*n*	%	(95 % CI)	*n*	%	(95 % CI)	*n*	%	(95 % CI)	*P* ^a^
**Diagnosis by/in**										
Paediatrician	454	49.0	(45.7 – 52.3)	215	41.5	(37.3 – 45.9)	211	58.0	(52.7 – 63.0)	< 0.001
Mental health professional	338	34.7	(31.7 – 37.9)	217	40.9	(36.7 – 45.3)	103	27.6	(23.2 – 32.5)	< 0.001
General practitioner	79	9.5	(7.7 –11.7)	48	10.4	(7.9 –13.6)	30	9.4	(6.7 –13.2)	0.663
Inpatient care	30	2.7	(1.9 – 3.9)	21	3.5	(2.3 – 5.4)	7	1.5	(0.7 – 3.2)	0.055
Other medical specialists	37	4.1	(3.0 – 5.6)	17	3.6	(2.2 – 5.8)	13	3.5	(2.0 – 6.0)	0.943
**Utilisation of therapeutic service providers**										
Occupational therapy	199	21.2	(18.6 – 23.9)	109	21.2	(17.8 – 25.1)	74	20.0	(16.2 – 24.5)	0.671
Speech therapy	65	7.0	(5.5 – 8.8)	31	6.3	(4.4 – 8.8)	29	7.5	(5.3 –10.7)	0.469

*n* = unweighted, % = weighted, CI = confidence interval, ADHD = attention-deficit/hyperactivity disorder

^a^p-values refer to the group comparisons for the parent report of the ADHD diagnosis

**Table 3:** Frequencies of utilisation of medical specialists and utilisation of therapeutic service providers in the past twelve months in the epidemiological survey data (total and by presence of a parent-reported ADHD diagnosis). Source: INTEGRATE-ADHD

	Online sample									
				Parent-reported ADHD diagnosis						
	Total			Present			Not present			
	*n*	%	(95 % CI)	*n*	%	(95 % CI)	*n*	%	(95 % CI)	*p* ^a^
**Utilisation of medical specialists^b^**										
Paediatrician	3,585	64.6	(63.3 – 65.9)	2,549	63.2	(61.6 – 64.7)	890	69.5	(66.8 – 72.0)	< 0.001
Mental health professional	2,954	50.4	(49.1 – 51.8)	2,571	62.9	(61.4 – 64.5)	288	21.5	(19.3 – 23.8)	< 0.001
General practitioner	1,836	34.5	(33.2 – 35.8)	1,346	35.0	(33.5 – 36.6)	407	33.1	(30.5 – 35.8)	0.225
**Utilisation of therapeutic service providers^b^**										
Occupational therapy	906	16.6	(15.6 –17.6)	671	17.1	(16.0 –18.4)	187	14.5	(12.7 –16.6)	0.029
Speech therapy	403	7.5	(6.8 – 8.2)	270	6.9	(6.1 – 7.7)	110	8.7	(7.2 –10.4)	0.032

*n* = unweighted, % = weighted, CI = confidence interval

^a^*p*-values refer to the group comparisons for the parent report of the ADHD diagnosis

^b^Multiple answers possible

**Table 4:** Results of adjusted multivariate binary-logistic regression analyses of administrative data (incidence cohort) for specialist diagnoses and utilisation of therapeutic service providers, and of online survey data for utilisation of medical specialists and utilisation of therapeutic service providers. Source: INTEGRATE-ADHD

Incidence cohort (administrative data) (*n* = 827)	Model 1			Model 2			Model 3		
	OR	(95 % CI)	*p*	OR	(95 % CI)	*p*	OR	(95 % CI)	*p*
**Diagnosis by**									
Paediatrician	1.58	(0.65 – 3.84)	0.313	–	–	–	1.59	(0.65 – 3.90)	0.309
Mental health professional	**2.70**	**(1.09 – 6.68)**	**0.032**	–	–	–	**2.78**	**(1.12 – 6.94)**	**0.028**
General practitioner	1.74	(0.62 – 4.89)	0.289	–			1.77	(0.62 – 5.00)	0.283
**Utilisation of therapeutic service providers**									
Occupational therapy	–	–	–	1.25	(0.80 –1.96)	0.329	1.35	(0.86 – 2.13)	0.190
Speech therapy	–	–	–	0.75	(0.39 –1.46)	0.402	0.79	(0.39 –1.60)	0.518
**Online sample (*n* = 4,798)**	**Model 1**			**Model 2**			**Model 3**		
	**OR**	**(95 % CI)**	***p***	**OR**	**(95 % CI)**	***p***	**OR**	**(95 % CI)**	***p***
**Utilisations of medical specialists**									
Paediatrician	**0.69**	**(0.55 – 0.87)**	**0.001**	*–*	*–*	*–*	**0.69**	**(0.55 – 0.86)**	**0.001**
Mental health professional	**3.04**	**(2.50 – 3.70)**	**< 0.001**	*–*	*–*	*–*	**2.99**	**(2.45 – 3.63)**	**< 0.001**
General practitioner	**0.79**	**(0.64 – 0.97)**	**0.024**	*–*	*–*	*–*	**0.79**	**(0.64 – 0.97)**	**0.024**
**Utilisation of therapeutic service providers**									
Occupational therapy	–	–	–	**1.54**	**(1.19 –1.97)**	**0.001**	**1.42**	**(1.09 –1.83)**	**0.008**
Speech therapy	–	–	–	0.92	(0.67 –1.26)	0.608	0.92	(0.67 –1.28)	0.638

OR = Odds Ratio, CI = confidence interval, CASMIN = Comparative Analysis of Social Mobility in Industrial Nations, FBB-ADHS = proxy rating (‘Fremdbeurteilungsbogen’), ADHD = attention-deficit/hyperactivity disorder, bold = significant results; All models controlled for gender, age at the time of the survey, parental education according to CASMIN classification (person with highest education in the household), migration background (two-sided), symptom severity (FBB-ADHS scale), prescription of ADHD medication (yes/no), parent-rated health status of the child, urbanicity, density of care (mean value of the quintile of regional ratio of the respective medical specialist group per 100,000 inhabitants in the spatial planning region of the child’s place of residence) for medical psychotherapists, child and adolescent psychiatrists, paediatricians and general practitioners.
